# Virus Control of Trafficking from Sorting Endosomes

**DOI:** 10.1128/mBio.00683-18

**Published:** 2018-07-24

**Authors:** Sebastian Zeltzer, Carol A. Zeltzer, Suzu Igarashi, Jean Wilson, Julie G. Donaldson, Felicia Goodrum

**Affiliations:** a Department of Cellular and Molecular Medicine, University of Arizona, Tucson, Arizona, USA; b BIO5 Institute, University of Arizona, Tucson, Arizona, USA; c National Heart, Lung and Blood Institute, National Institutes of Health, Bethesda, Maryland, USA; d Department of Immunobiology, University of Arizona, Tucson, Arizona, USA; e University of Arizona Center on Aging, Tucson, Arizona, USA; Princeton University

**Keywords:** ARF6, TRE17, USP6, cytomegalovirus, endocytic trafficking, endosomes, herpesviruses

## Abstract

The maintenance of cell surface proteins is critical to the ability of a cell to sense and respond to information in its environment. As such, modulation of cell surface composition and receptor trafficking is a potentially important target of control in virus infection. Sorting endosomes (SEs) are control stations regulating the recycling or degradation of internalized plasma membrane proteins. Here we report that human cytomegalovirus (HCMV), a ubiquitous betaherpesvirus, alters the fate of internalized clathrin-independent endocytosis (CIE) cargo proteins, retaining them in virally reprogrammed SEs. We show that the small G protein ARF6 (ADP ribosylation factor 6), a regulator of CIE trafficking, is highly associated with SE membranes relative to uninfected cells. Combined with the observation of accumulated CIE cargo at the SE, these results suggest that infection diminishes the egress of ARF6 and its cargo from the SE. Expression of ubiquitin-specific protease 6 (USP6), also known as TRE17, was sufficient to restore ARF6 and some ARF6 cargo trafficking to the cell surface in infected cells. The USP activity of TRE17 was required to rescue both ARF6 and associated cargo from SE retention in infection. The finding that TRE17 expression does not rescue the trafficking of all CIE cargos retained at SEs in infection suggests that HCMV hijacks the normal sorting machinery and selectively sorts specific cargos into endocytic microdomains that are subject to alternative sorting fates.

## IMPORTANCE

Cells maintain their surface composition, take up nutrients, and respond to their environment through the internalization and recycling of cargo at the cell surface through endocytic trafficking pathways. During infection with human cytomegalovirus (HCMV), host endocytic membranes are reorganized into a juxtanuclear structure associated with viral assembly and egress. Less appreciated is the effect of this reorganization on the trafficking of host proteins through the endocytic pathway. We show that HCMV retains internalized cargo and the effector of clathrin-independent endocytosis at sorting endosomes. The retention of some cargo, but not all, was reversed by overexpression of a ubiquitin-specific protease, TRE17. Our results demonstrate that HCMV induces profound reprogramming of endocytic trafficking and influences cargo sorting decisions. Further, our work suggests the presence of a novel ubiquitin-regulated checkpoint for the recycling of cargo from sorting endosome. These findings have important implications for host signaling and immune pathways in the context of HCMV infection.

## INTRODUCTION

The ability of a cell to sense changes in its environment, to transmit signals inward, and to respond to these changes directly influences its state of differentiation, activation, stress, proliferation, and survival. Detection of and responses to the bulk of external cues are mediated by an array of plasma membrane receptors and other cell surface proteins. On short time scales, the surface concentration of these proteins is regulated by endocytosis and subsequent sorting decisions that culminate in either protein degradation or recycling back to the plasma membrane (PM). This binary decision (to recycle or to degrade) is executed in sorting endosomes (SE), structures that act as platforms capable of broadly tuning the ability of a cell to respond to a milieu of signals (1).

Human cytomegalovirus (HCMV), a betaherpesvirus, is notable for its reorganization of the host secretory membranes into a juxtanuclear, virus-induced organelle (2–5). In this structure, the Golgi apparatus is organized into a ring-like structure with various endocytic and exocytic vesicles at the center (4, 5). Viral structural proteins are associated with membranes in this region, and, as such, this dramatic reorganization of host membranes has been correlated with the assembly and egress of viral particles and termed the viral assembly compartment (VAC) (2–6). In recent years, however, a number of host proteins not thought to be related to viral assembly and egress have been found localized to this compartment and its associated membranes, including mTOR, β-catenin, transferrin (TF) receptor (TFR), and epidermal growth factor receptor (EGFR) (5, 7–10). These observations suggest that the virus alters vesicular trafficking more broadly, likely affecting host cell trafficking and signaling; however, the extent of the effect and the significance of the manner in which infection alters host trafficking are unknown.

To better understand the implications and extent of virally manipulated endocytic trafficking, we mapped infection-induced alterations to cargo endocytosis and trafficking during HCMV infection. We focused on the ARF6 (ADP ribosylation factor 6)-associated clathrin-independent endocytosis (CIE) pathway, an immunologically relevant endocytic pathway regulating the internalization and recycling of molecules such as major histocompatibility complex class I (MHC-I) and MHC-II, interleukin receptor 2 subchain alpha (ILR2-α), and the CD59 complement protein, as well as migratory and adhesion proteins such as CD147, β1-integrin, and E-cadherin (11–14). We found that infection with HCMV results in the aberrant accumulation and retention of both the CIE regulator ARF6 and CIE cargos in enlarged SEs, representing a fate that is shared with clathrin-dependent endocytic (CDE) cargo. This retention appears to be the result of delayed recycling from the SE. Overexpression of host ubiquitin-specific protease 6 (USP6), referred to here as TRE17, is sufficient to reverse the retention of ARF6 and of some, but not all, CIE cargos in the context of infection. For example, MHC-I and CD98 were insensitive to TRE17-mediated trafficking in the context of infection. As TRE17 functions to stimulate ARF6 and CIE cargo recycling to the cell surface, this result suggests that HCMV alters cargo-specific sorting decisions resulting in some cargo being refractory to TRE17-mediated sorting. Lastly, we found that the ability of TRE17 to facilitate ARF6/CIE cargo recycling was dependent on its USP activity, implying new regulatory importance for USPs in vesicular trafficking. This work charts a new mechanism by which HCMV manipulates the host cell, rewiring decision-making at SEs. Further, our findings implicate TRE17 in the dissociation of ARF6 membranes from EEA1 sorting endosomes in a ubiquitin-specific protease-dependent manner.

## RESULTS

### HCMV induces the retention of CIE cargos in the SE.

While HCMV relocalizes a number of signaling proteins to the VAC during productive infection in fibroblasts (5, 7–10, 15), it is not known how the virus alters trafficking from the cell surface to affect this relocalization. We sought to understand how CIE was altered by infection since CIE governs the trafficking of proteins known to be affected by HCMV and by a number of immune regulators, such as MHC-I and CD59. While similar to CDE in that CIE cargo can traffic through SEs, CIE trafficking is not entirely restricted to passage through the SE (16), and many cargos have only a transient association with the SE.

**FIG 1:**
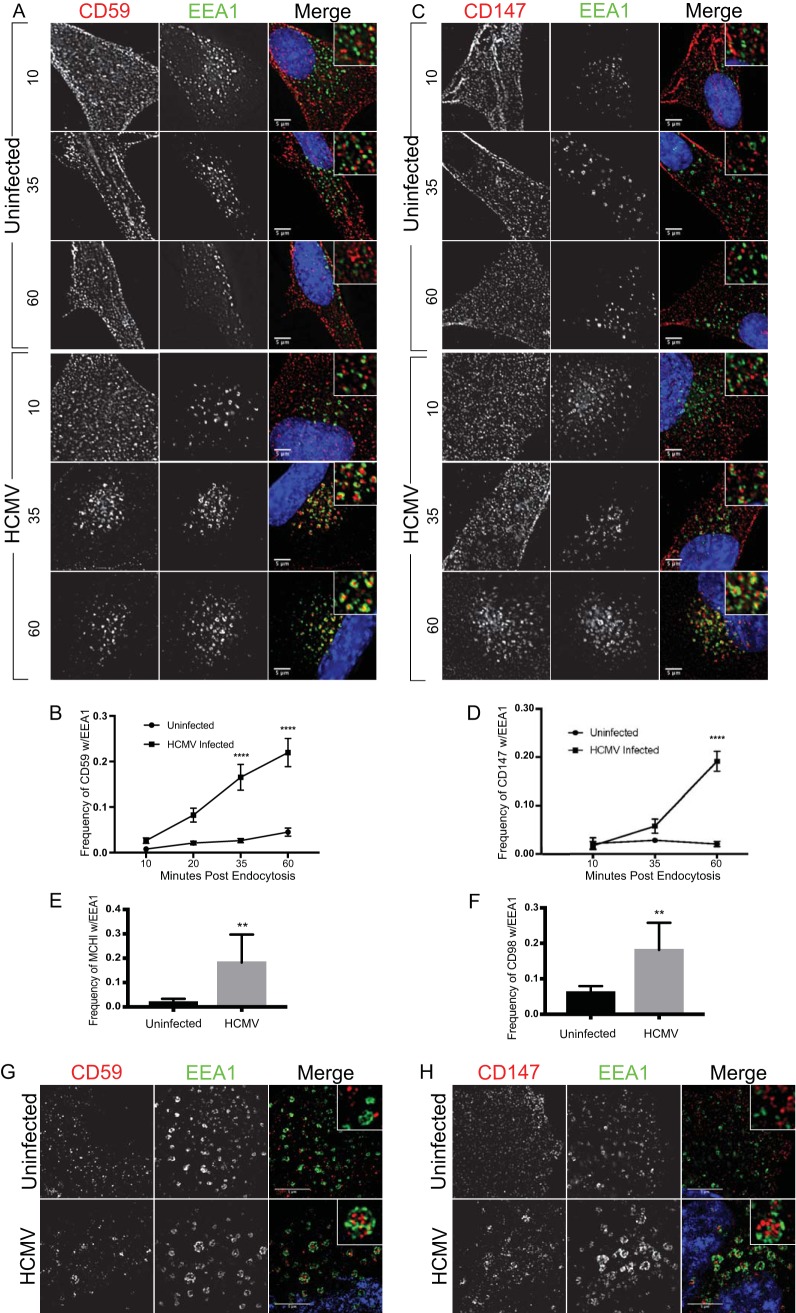
Infection with HCMV results in retention of CIE cargo at sorting endosomes. Fibroblasts were mock infected or infected with HCMV-TB40/E at an MOI of 1. At 48 hpi, mouse monoclonal antibodies against CD59 (A, B, and G), CD147 (C, D, and H), MHC-I (E), or CD98 (F) were incubated with cells to label surface proteins. Cells were fixed at the indicated time points (minutes) following internalization of antibody/cargo. (A and C) Time in minutes postinternalization is indicated to the left of corresponding panels. (G and H) Cells were fixed at 60 min postinternalization. Internalized CIE cargo was detected with an anti-mouse Alexa Fluor 647 secondary antibody (red). Endogenous EEA1 was indirectly visualized by labeling with anti-EEA1 rabbit antibody and secondary anti-rabbit Alexa Fluor 546 (green). Infection was confirmed by detection of virally expressed GFP (not shown). A merged image with a higher-magnification inset of each channel with DAPI (4′,6-diamidino-2-phenylindole) staining to indicate nuclei is shown on the right of each panel. Results of the experiments represented in panels A and C were imaged using a DeltaVision deconvolution microscope. Results of the experiments represented in panels G and H were imaged using SIM. Each image corresponds to the same representative focal plane. Bar, 5 µm. (B, D, E, and F) Image quantification was performed by the use of the Squassh workflow method in the Mosaic suite of ImageJ and Fiji. The *y*-axis data represent the frequency of coincidence of each marker with EEA1 and are averages of results from three independent experiments. Values represent means ± standard deviations (SEM); an average of 10 cells were analyzed per time point. Quantifications of results from the experiments represented in panels A and C are shown in panels B and D, respectively. Data from experiments E and F are displayed as quantifications only (see Fig. S1B and C for the respective images). Statistical significance was determined by a one-way analysis of variance (ANOVA) with Tukey corrections. Asterisks (**, *P* < 0.01; ****, *P* < 0.0001) represent statistically significant differences determined in three or more independent experiments.

CD59 is a classic marker of CIE that can pass through the SE, where it is sorted for recycling to the cell surface or for degradation (13). We monitored the endocytosis of CD59 using an antibody uptake assay combined with indirect immunofluorescence against the endogenous SE marker EEA1 over a time course (10, 35, and 60 min postuptake). Because the HCMV-encoded Fc receptor, gp68, has high affinity for human and rabbit polyclonal IgG antibodies (17, 18), we used only mouse monoclonal antibodies, which have no affinity toward gp68 (17). In uninfected primary lung fibroblasts, regardless of the time postendocytosis, we found only minor populations of CD59 coincident with EEA1 (Fig. 1A). However, during HCMV infection, CD59 accumulated in the SE as indicated by the increased association with EEA1 over time postuptake. The association of CD59 with EEA1 over time in multiple independent experiments is quantified in Fig. 1B. Of note, the EEA1-positive endosomes were enlarged in the context of infection (up to 1 µm), consistent with the observations of other groups (5, 19).

10.1128/mBio.00683-18.1 FIG S1  HCMV infection results in retention of CIE cargo at sorting endosomes. (A) Fibroblasts were infected with HCMV-TB40/E at an MOI of 1. At 48 hpi, mouse monoclonal antibodies against CD147 were incubated with cells to label surface proteins. Cells were fixed at 240 min postinternalization of antibody/cargo (indicated to the left of the panel). Internalized CIE cargo was detected with anti-mouse Alexa Fluor 647 (red). Endogenous EEA1 was indirectly visualized by labeling with anti-EEA1 rabbit antibody and secondary anti-rabbit Alexa Fluor 546 (green). Infection was confirmed by detection of virally expressed GFP (not shown). A merged image with a higher-magnification inset of each channel with DAPI staining to indicate nuclei is shown on the right of each panel. Panels B and C correspond to Fig. 1E and F, respectively (see the Fig. 1 legend for details). (D to G) Fibroblasts were mock infected or infected with HCMV TB40/E at an MOI of 1. At 48 h postinfection, mouse monoclonal antibody against CD59 (D) or CD147 (F) with TF_546_ at 100 µg/ml was incubated with cells to label surface proteins. Cells were fixed following internalization of antibody/cargo (time points [in minutes] are indicated at left of each panel). (A, D, and F) Internalized CIE cargo was detected with anti-mouse Alexa Fluor 647 (red). (A) Endogenous EEA1 was indirectly visualized by labeling with anti-EEA1 rabbit antibody and secondary anti-rabbit Alexa Fluor 546 (green). (D and F) TF_546_ was visualized via its own (green) fluorescence. A merged image with a higher-magnification inset of each channel with DAPI staining to indicate nuclei is shown on the right of each panel. The experiments represented in panels A, D, and F were imaged using a DeltaVision deconvolution microscope. Each image corresponds to the same representative focal plane. Bar, 5 µm. (E and G) Quantifications of data from the experiments represented in panels D and F are shown in panels E and G, respectively. Image quantification was performed by the use of the Squassh workflow method in the Mosaic suite of ImageJ and Fiji. The *y*-axis data represent the frequency of coincidence of each marker with EEA1 or TF_546_. The values in panels E and G represent means ± SEM of results of experiments in which an average of 7 cells were analyzed per condition. Statistical significance was determined by a one-way ANOVA with Tukey corrections. Asterisks (*, *P* values = <0.05; ****, *P* values = <0.0001) represent statistically significant differences. Download FIG S1, EPS file, 2.3 MB. Copyright © 2018 Zeltzer et al. 2018 Zeltzer et al. This content is distributed under the terms of the Creative Commons Attribution 4.0 International license.

There is a separate class of alternative CIE markers that includes CD147, CD98, and CD44, all of which have been described to traffic via routes that do not necessarily include SEs (16). Therefore, we analyzed the association of the alternative CIE cargo, CD147, with EEA1 following its internalization using an antibody uptake assay. As anticipated, CD147 was largely unassociated with EEA1 at all times postinternalization in uninfected cells (Fig. 1C). In sharp contrast, the frequency of coincidence of CD147 with EEA1 increased over time during infection similarly to the CD59 results. The results of quantitation of CD147-EEA1 association in multiple experiments are shown in Fig. 1D. We observed retention of CD147 cargo in EEA1-positive endosomes for up to 4 h, representing the latest time point postinternalization that we have tested (see Fig. S1A in the supplemental material). Two additional CIE cargos, the classical cargo MHC-1 (Fig. 1E) and the alternative cargo CD98 (Fig. 1F), also showed increased association with the SE in cells infected with HCMV. Representative images of MHC-I and CD98 are shown in Fig. S1B and C.

Of note, MHC-I and CD59 exhibited lower association with SEs in uninfected cells than had been previously observed in cell lines (13, 20). Their association with EEA1 appeared consistent with that described for two alternative cargos, CD147 and CD98, that have a predilection to recycle. This suggests that the CIE cargos tested may exhibit higher recycling rates in primary lung fibroblasts.

In HCMV-infected cells, the staining of EEA1 on the SE membranes appeared to be discontinuous and was often distinct from the associated cargo (Fig. 1A and C). To gain greater resolution of the CD59 and CD147 data relative to EEA1, we used superresolution structured illumination microscopy (SIM) to examine EEA1^+^/CD59^+^ and EEA1^+^/CD147^+^ vesicles at 60 min postuptake. Not surprisingly, in uninfected cells at 60 min postendocytosis, we found that CD59 (Fig. 1G) and CD147 (Fig. 1H) were mostly present in discrete structures relative to EEA1. These results indicate that HCMV biases both classical and alternative CIE cargo trafficking toward the SE where they are retained.

### Infection merges clathrin-independent and -dependent endocytic sorting pathways.

While the CIE and CDE pathways represent distinct routes of endocytosis, cargo from both pathways can converge at SEs—a node connecting the two pathways (1, 20). The prototypical CDE cargo, transferrin (TF), localizes with EEA1^+^ SEs at the VAC in HCMV-infected cells (4, 9, 15). Therefore, we analyzed the trafficking and association of CIE and CDE cargo in infection. We analyzed the internalization and trafficking of a classical CIE marker, CD59, relative to the classical CDE marker, transferrin receptor (TFR), using antibody-stimulated uptake of CD59 and fluorescently conjugated transferrin (TF_546_). In uninfected cells, the coincidence of CD59 with TF_546_ was seen in only rare cases (frequency of ~8% by 60 min) (Fig. S1D; quantified in panel E). HCMV infection increased the frequency of coincidence of CD59 with TF_546_ to ~20% and 25% at 35 and 60 min postinternalization, respectively. Similarly, CD147 also showed an increased association with TF_546_ in HCMV-infected cells relative to uninfected cells at 60 min postinternalization (Fig. S1F and G). The increased coincidence of CDE and CIE cargo over time suggests that HCMV has merged CIE and CDE pathways and induces a shared fate of retention at the SE, which stands in striking contrast to vesicular trafficking in uninfected cells.

### HCMV increases the association of ARF6 with EEA1.

**FIG 2:**
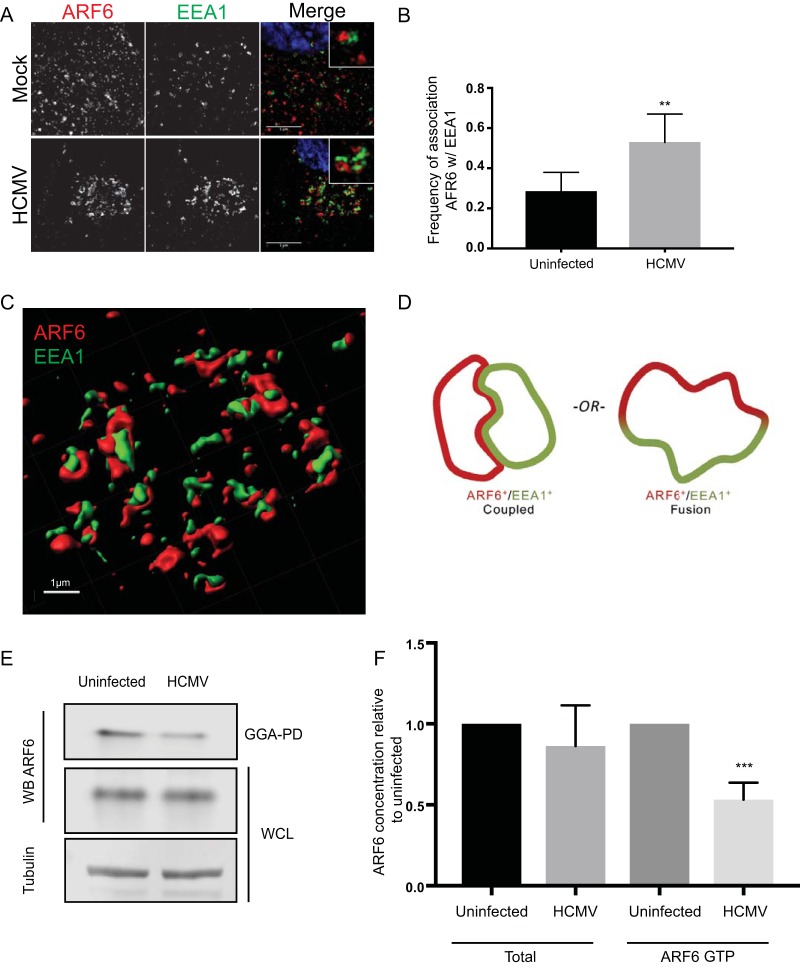
Infection with HCMV results in increased EEA1-ARF6 association and decreased ARF6 GDP-GTP cycling. (A and B) Fibroblasts were transfected with plasmid expressing ARF6_mRuby_. At approximately 24 h posttransfection, cells were mock infected or infected with HCMV-TB40/E dark at an MOI of 1. At 48 hpi, cells were fixed. (A) Endogenous EEA1 was indirectly visualized by labeling with anti-EEA1 rabbit antibody and secondary anti-rabbit Alexa Fluor 488 (green). Exogenous ARF6_mRuby_ was visualized via its own fluorescence (red). Infection was confirmed by VAC morphology. A merged image with a higher-magnification inset of each channel is shown on the right of each panel where DAPI (blue) was used to stain nuclei. Cells were imaged using SIM. Each image corresponds to the same representative focal plane. Bar, 5 µm. (B) Image quantification was performed by the use of the Squassh workflow method in the Mosaic suite of ImageJ and Fiji. The *y*-axis data represent the frequency of association of ARF6 with EEA1 and were calculated by hand-counting the number of plasma-membraned independent (vesicular) ARF6-positive structures separated by <1 px from EEA1-positive structures. Each value was divided by the total number of ARF6 vesicles to calculate the percentage of ARF6 associated with EEA1. The values in panel B represent means ± standard deviations (SD) of results from three independent experiments in which an average of 14 cells were analyzed per condition. (C) Imaris projection of ARF6-EEA1 heterotypic structures rendered from the SIM image in panel A. (D) Model representing possible ARF6/EEA1 structures in infection: coupled vesicles or fused vesicles. (E) Fibroblasts were mock infected or infected with HCMV-TB40/E at an MOI of 1. At 48 hpi, ARF6-GTP was pulled down from cell lysates (GGA-PD). Whole-cell lysates (WCL) were probed for total ARF6 and tubulin as a loading control. (F) WCL ARF6 was normalized to tubulin and uninfected levels. GGA-PD ARF6 (ARF6 GTP) was normalized to uninfected GGA-PD ARF6 levels. Data represent means ± SD of results from three separate experiments. Statistical significance was determined by a one-way ANOVA with Tukey corrections. Asterisks (**, *P* values = <0.01; ***, *P* values = <0.001) represent statistically significant differences.

Our observations that CIE cargo is retained in SEs of infected cells revealed a consistent feature: EEA1 labeling on endosomes was rarely contiguous. Domains of SEs lacked EEA1, and at times these EEA1-negative regions were occupied by CIE cargo (Fig. 1G and H). On the basis of this, we reasoned that these domains might represent regions where ARF6-positive vesicles carrying CIE cargo were interacting with SEs. ARF6 is a guanosine triphosphate (GTP)-binding protein that can modulate the endocytosis of CIE cargo and subsequent recycling events (21). Following endocytosis, ARF6-positive vesicles transiently associate with SEs to deposit cargo (16, 20, 21). To determine if infection alters the frequency of this association, we analyzed ARF6 and EEA1 localization under steady-state conditions in both uninfected and HCMV-infected cells. To assist us in visualizing ARF6, we generated an expression construct where ARF6 was fused to mRuby2 (ARF6_mRuby_). In HCMV-infected cells, there was a marked increase in the proportion of vesicular ARF6_mRuby_ associated with EEA1 relative to uninfected cells (Fig. 2A). Approximately 55% of vesicular ARF6 was associated with EEA1 in infected cells compared to ~25% in uninfected cells (Fig. 2B). To ensure that the enhanced ARF6-EEA1 association seen in infection was not merely a consequence of endocytic concentration at the VAC, we analyzed ARF6-EEA1 association in infected cells with endocytic pools ranging in size from ~160 µm^2^ to ~650 µm^2^ and found the ARF6-EEA1 association to be independent of the endocytic concentration (Fig. S2).

10.1128/mBio.00683-18.2 FIG S2  ARF6-EEA1 association is independent of vesicular concentration. (A) Fibroblasts were transfected with plasmid expressing ARF6_mRuby_. At approximately 24 h posttransfection, cells were infected with HCMV-TB40/E dark at an MOI of 1. At 48 hpi, cells were fixed. Endogenous EEA1 was indirectly visualized by labeling with anti-EEA1 rabbit antibody and secondary anti-rabbit Alexa Fluor 488 antibody (green). Exogenous ARF6_mRuby_ was visualized via its own fluorescence (red). Infection was confirmed by VAC morphology. A merged image with a higher-magnification inset of each channel is shown on the right of each panel. The top and bottom panels represent infected cells with planer VAC areas of ~159 µm^2^ and 459 µm^2^, respectively. The results of the experiments represented in panel A were imaged using a DeltaVision deconvolution microscope. Each image corresponds to the same representative focal plane. Bar, 5 µm. (B) Image quantification was performed by analyzing 11 cells for ARF6-EEA1 association versus VAC area (in square micrometers). Regions of interest (ROIs) were drawn around endosomal pools of infected cells. The VAC area was calculated from ROIs using the Measure tool in ImageJ. The *y*-axis data in panel B represent frequencies of ARF6 association with EEA1 and were calculated from each image’s corresponding ROI. Calculations were made by hand-counting the number of plasma-membrane-independent (vesicular) ARF6-positive structures separated by <1 px from EEA1-positive structures. Each value was divided by the total number of ARF6 vesicles to calculate a percentage of ARF6 associated with EEA1. Each dot represents values from an individual cell. Cells from panel A are identified by arrows in panel B. Download FIG S2, EPS file, 2.4 MB. Copyright © 2018 Zeltzer et al. 2018 Zeltzer et al. This content is distributed under the terms of the Creative Commons Attribution 4.0 International license.

The coassociation of ARF6 and EEA1 in both uninfected and HCMV-infected cells was rarely represented by overlapping colocalizations. Instead, the markers appeared to label discrete domains (Fig. 2A). Imaris rendering of these SIM images further details the distinct EEA1 and ARF6 domains evident in SEs associated with HCMV infection (Fig. 2C). This coassociation without colocalization was highly reminiscent of our observations of CIE cargo with EEA1 (Fig. 1G and H). It is unclear whether the ARF6/EEA1 vesicles in infections represent a coupling of distinct vesicles or genuine fusion events where the lumen is shared, but EEA1 and ARF6 domains remain distinct (Fig. 2D). However, because ARF6-EEA1 vesicles are seen in uninfected cells (Fig. 2A and B), these structures likely reflect a normal albeit minor (~25%) part of cell biology that is enhanced by HCMV infection. Heterotypic ARF6/EEA1 vesicles were also enlarged in HCMV-infected cells compared to uninfected cells, suggesting that infection exaggerates this structure. This discovery offers a new tool for studying the normally transient association of ARF6 and EEA1 endocytic sorting pathways.

### Infection reduces ARF6 activation.

Following endocytosis, CIE cargos are often recycled to the PM in an ARF6-dependent manner (20–22). Indeed, alternative CIE cargos such as CD147 show enhanced recycling through ARF6-Rab22a-positive tubules relative to classical cargos (16, 23). We therefore wondered if the increased association of vesicular ARF6 and CIE cargos with SEs might result from decreased ARF6 recycling. ARF6 cycles between plasma membrane and endosomal states in a GTP-to-GDP-dependent manner (21, 24, 25). Following endocytosis, ARF6-GTP is hydrolyzed and inactivated by GTPase-activating proteins (GAPs) at structures such as the SE (26) and GTP hydrolysis is required for ARF6 to recycle back to the PM (21, 27). Therefore, we measured ARF6 activation in both infected and uninfected cells using ARF6-GTP pulldown assays. ARF6-GTP levels were diminished in infected cells (Fig. 2E and F). Since ARF6 is always bound to either GTP or GDP, the diminished ARF6-GTP levels are consistent with an increased level of vesicular ARF6-GDP and a block in ARF6 recycling underlying the accumulation of cargo at the SE.

### Ubiquitin-specific protease 6 restores trafficking of some CIE cargos and ARF6 from the SE.

**FIG 3:**
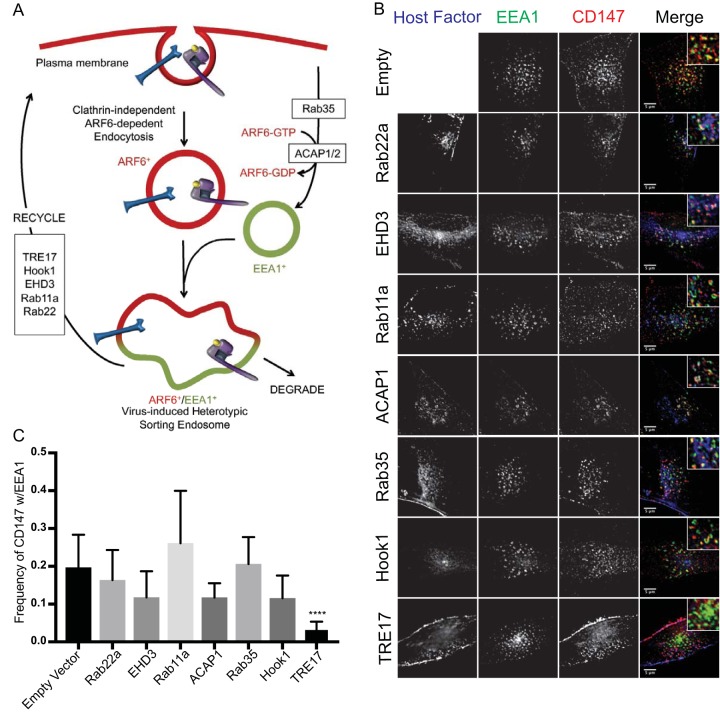
Screen of host recycling factors reveals role for TRE17 in reversing CD147 endosomal retention in HCMV-infected cells. (A) Model of HCMV-altered CIE. The candidate host factors analyzed in panel B are boxed. (B) Fibroblasts were transfected with plasmid expressing host factor or an empty vector control. At approximately 24 h posttransfection, cells were infected with HCMV-TB40/E dark at an MOI of 1. At 48 hpi, mouse monoclonal antibodies against CD147 were incubated with cells to label surface proteins. Cells were fixed at 60 min following internalization of antibody/cargo. Internalized CD147 was detected with anti-mouse Alexa Fluor 647 (red). Endogenous EEA1 was indirectly visualized by labeling with anti-EEA1 rabbit antibody and secondary anti-rabbit Alexa Fluor 546 (green). Infection was confirmed by VAC morphology. Host factors were detected by labeling against respective epitope tags or by directly imaging fluorescent proteins (blue). A merged image with a higher-magnification inset of each channel is shown on the right of each panel. Results of the experiments performed as described for panel B were imaged using a DeltaVision deconvolution microscope. Each image corresponds to the same representative focal plane. Bar, 5 µm. (C) Image quantification was performed by the use of the Squassh workflow method in the Mosaic suite of ImageJ and Fiji. The *y*-axis data represent the frequency of coincidence of each marker with EEA1 and represent averages of results from three independent experiments. The values in panel C represent means ± SD; an average of 20 cells were analyzed per condition. Statistical significance was determined by a one-way ANOVA with Tukey corrections. Asterisks (****, *P* values = <0.0001) represent statistically significant differences.

While no one specific host factor has been identified as regulating the dissociation of ARF6 membranes from EEA1-positive SEs for CIE cargo recycling, downregulation of numerous CIE regulatory proteins (Fig. 3A) results in phenotypes reminiscent of HCMV-induced retention of CIE cargo in the SE (9, 22, 26, 28–30). Further, a number of these CIE regulators, including Rab11a, Hook1, ACAP1/2, and EHD3, are targeted by microRNAs or downregulated by infection with HCMV (15, 31). We therefore screened known regulators of CIE cargo recycling to determine if their exogenous expression could rescue the recycling of the alternative CIE marker, CD147, from the SE in HCMV-infected cells. We analyzed CD147 association with EEA1 at 60 min postendocytosis of antibody bound to cargo in cells infected with HCMV and overexpressing one of the CIE regulators listed in Fig. 3A and B or an empty vector control. Using this approach, overexpression of the ARF6 GTPase-activating protein (GAP), ACAP1, or its transporter Rab35 had no effect on the retention of CD147 at the SE (Fig. 3B and C). Further, factors required for recycling, including the tubular endosome stabilizing protein EHD3 and the endocytic recycling complex proteins Rab11a and Rab22a, had no effect on CD147 retention in the SE (22, 28, 32). Expression of Hook1, an adapter protein that facilitates CIE cargo recycling from early endosomes, also had no effect on CD147 retention (22). However, overexpression of the USP and ARF6 regulator, TRE17/USP6, alleviated CD147 retention at the SE. Exogenous expression of TRE17 significantly reduced the association of CD147 with SEs from the ~20% level seen with empty vector to ~3%, similar to the association in uninfected cells (Fig. 3C). These results indicate that overexpression of TRE17 restores trafficking of CIE cargo from the SE.

**FIG 4:**
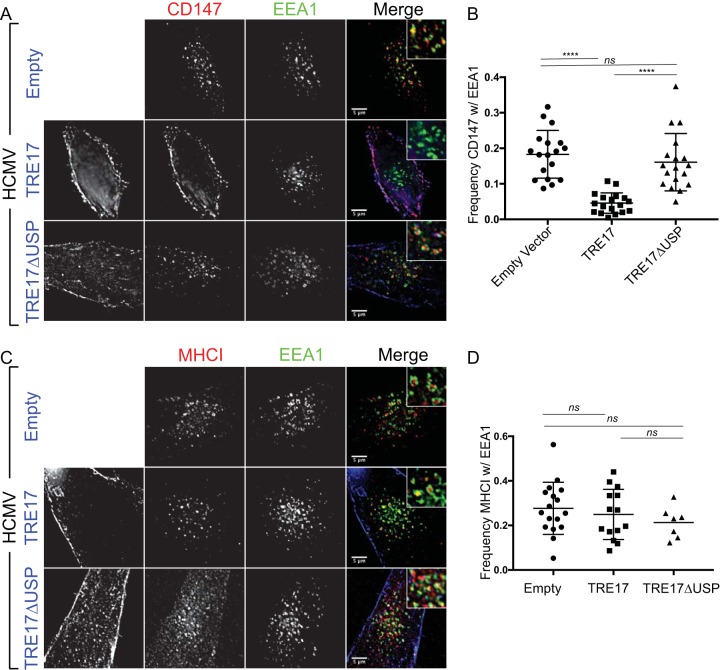
TRE17 reduces CD147 retention at SEs in a ubiquitin-specific protease-dependent manner. (A and C) Fibroblasts were transfected with plasmids expressing TRE17_GFP_, TRE17ΔUSP_GFP_, or empty vector. At 24 h posttransfection, cells were infected with HCMV-TB40/E dark at an MOI of 1. At 48 hpi, mouse monoclonal antibodies against CD147 (A) or MHC-I (C) were incubated with cells to label surface proteins. Cells were fixed at 60 min following internalization of antibody/cargo. Internalized CIE cargo was detected with anti-mouse Alexa Fluor 647 (red). Endogenous EEA1 was indirectly visualized by labeling with anti-EEA1 rabbit antibody and secondary anti-rabbit Alexa Fluor 546 (green). TRE17_GFP_ or TRE17ΔUSP_GFP_ was visualized via its own fluorescence (blue). Infection was confirmed by VAC morphology. A merged image with a higher-magnification inset of each channel is shown on the right of each panel. Results of the experiments performed as described for panels A and C were imaged using a DeltaVision deconvolution microscope. Each image corresponds to the same representative focal plane. Bar, 5 µm. (B and D) Image quantification was performed by the use of the Squassh workflow method in the Mosaic suite of ImageJ and Fiji. The *y*-axis data represent the frequency of coincidence of CIE cargo with EEA1 and are averages of results from three independent experiments. Quantifications of data from the experiments performed as described for panels A and C are shown in panels B and D, respectively. The values in panel B represent means ± SD; an average of 18 cells were analyzed per condition. Each dot represents an individual cell’s CD147-EEA1 association frequency. The values in panel D represent means ± SD; 7 to 17 cells were analyzed per condition. Statistical significance was determined by a one-way ANOVA with Tukey corrections. Asterisks (***, *P* values = <0.001; ****, *P* values = <0.0001) represent statistically significant differences. ns, not significant.

TRE17 counteracts membrane-associated RING-CH (MARCH) E3 ubiquitin ligase-dependent targeting of CIE cargos to the lysosome via its USP activity and has been shown to deubiquitylate the CIE cargo CD98 and rescue the effects of MARCH expression on surface levels of both CD98 and MHC-I (30). We hypothesized that the USP activity of TRE17 may underlie its ability to restore CIE cargo trafficking from the SE in the context of infection. To test this, we expressed a TRE17 variant where the USP domain was disrupted by a cysteine-to-serine point mutation at amino acid 541, TRE17_C541S_ (TRE17ΔUSP) (30, 33). Relative to wild-type TRE17, TRE17ΔUSP failed to rescue CD147 trafficking, indicating a requirement for the USP activity of TRE17 in CD147 trafficking from the SE (Fig. 4A and B). To exclude the possibility that overexpression of TRE17 affected CD147 trafficking by preventing its endocytosis and, therefore, its passage to the SE, we examined infected cells overexpressing TRE17 green fluorescent protein (TRE17_GFP_) at 10 min and 30 min post-CD147 endocytosis. At 10 min postendocytosis, the majority of the CD147 signal was present in an EEA1^−^ endosomal pool near the VAC (Fig. S3). By 30 min postuptake, however, the majority of CD147 was associated with TRE17_GFP_ at the PM. Therefore, TRE17 was not preventing the internalization of CD147 but instead stimulated its recycling from the endosomal pool.

10.1128/mBio.00683-18.3 FIG S3  TRE17 mediates CD147 trafficking postinternalization. (A) Fibroblasts were transfected with plasmid expressing TRE17_GFP_ or empty vector. At approximately 24 h posttransfection, cells were infected with HCMV-TB40/E dark at an MOI of 1. At 48 hpi, mouse monoclonal antibodies against CD147 were incubated with cells to label surface proteins. Cells were fixed at indicated time points following internalization of antibody/cargo (time points are indicated in minutes at left of each panel). Internalized CIE cargo was detected with anti-mouse Alexa Fluor 647 (red). Endogenous EEA1 was indirectly visualized by labeling with anti-EEA1 rabbit antibody and secondary anti-rabbit Alexa Fluor 546 (green). TRE17_GFP_ was visualized via its own fluorescence (blue). Infection was confirmed by VAC morphology. A merged image with a higher-magnification inset of each channel is shown on the right of each panel. Results of experiments were imaged using a DeltaVision deconvolution microscope. Each image corresponds to the same representative focal plane. Bar, 5 µm. Download FIG S3, EPS file, 2.4 MB. Copyright © 2018 Zeltzer et al. 2018 Zeltzer et al. This content is distributed under the terms of the Creative Commons Attribution 4.0 International license.

To determine if TRE17 broadly restores the sorting of CIE cargo from the SE in HCMV-infected cells, we examined the effect of TRE17 overexpression on the association of MHC-I with EEA1. Surprisingly, MHC-I was largely unaffected by the expression of TRE17. These results were particularly surprising since TRE17 overexpression has been shown to increase surface levels of both MHC-I and CD98 (30). In uninfected cells, MHC-I was highly enriched at the PM of cells overexpressing TRE17 relative to an empty vector control (Fig. S4A), a finding that is consistent with previous work (30) and that confirms that TRE17 can indeed influence the trafficking of cargos such as MHC-I to the cell surface in our primary fibroblasts. We then analyzed the association of CD59 and CD98. Similarly to CD147, TRE17 expression resulted in diminished retention of CD59 in SEs of infected cells (Fig. S4B and C). However, CD98 was unaffected by TRE17, remaining retained at SEs (Fig. S4D and E) similarly to MHC-I. Further, TRE17 had no effect on the CDE cargo, TF_546_ (Fig. S5). This result suggests that infection results specifically in retention of cargo, such as MHC-I and CD98, at SEs and that TRE17 is not sufficient to rescue the virus-imposed block to their trafficking.

10.1128/mBio.00683-18.4 FIG S4  TRE17 expression selectively alters CIE trafficking. (A) Uninfected cells were transfected with TRE17_GFP_ or an empty vector. At 24 h posttransfection, mouse monoclonal antibodies against MHC-I were incubated with cells to label surface proteins. Cells were fixed at 30 min following internalization of antibody/cargo. Internalized CIE cargo was detected with anti-mouse Alexa Fluor 647 (red). Endogenous EEA1 was indirectly visualized by labeling with anti-EEA1 rabbit antibody and secondary anti-rabbit Alexa Fluor 546 (green). TRE17_GFP_ was visualized via its own fluorescence (blue). A merged image with a higher-magnification inset of each channel is shown on the right of each panel. Results of experiments were imaged using a DeltaVision deconvolution microscope. Each image corresponds to the same representative focal plane. Bar, 5 µm. An average of 15 cells were counted per condition. (B and D) Fibroblasts were transfected with TRE17_GFP_ or empty vector control. At approximately 24 h posttransfection, cells were infected with HCMV-TB40/E dark at an MOI of 1. At 48 hpi, mouse monoclonal antibodies against CD59 (B) or CD98 (D) were incubated with cells to label surface proteins. Cells were fixed at 60 min following internalization of antibody/cargo. Internalized CIE cargo was detected with anti-mouse Alexa Fluor 647 (red). Endogenous EEA1 was indirectly visualized by labeling with anti-EEA1 rabbit antibody and secondary anti-rabbit Alexa Fluor 546 (green). TRE17_GFP_ was visualized via its own fluorescence (blue). Infection was confirmed by VAC morphology. A merged image with a higher-magnification inset is shown on the right of each panel. Results of experiments were imaged using a DeltaVision deconvolution microscope. Each image corresponds to the same representative focal plane. Bar, 5 µm. (C and E) Quantifications of results from the experiments represented in panels B and D are shown in panels C and E, respectively. Image quantification was performed by the use of the Squassh workflow method in the Mosaic suite of ImageJ and Fiji. The *y*-axis data represent frequencies of association of CIE cargo with EEA1 and are averages of results from three experiments. The values in panels C and E represent means ± SD where an average of 15 cells were analyzed per condition. Each dot represents an individual cell’s CIE association with EEA1. Statistical significance was determined by a one-way ANOVA with Tukey corrections. Asterisks (**, *P* values = <0.01) represent statistically significant differences. Download FIG S4, EPS file, 2.6 MB. Copyright © 2018 Zeltzer et al. 2018 Zeltzer et al. This content is distributed under the terms of the Creative Commons Attribution 4.0 International license.

10.1128/mBio.00683-18.5 FIG S5  TRE17 has no effect on retained CDE cargo. Fibroblasts were transfected with plasmid expressing TRE17_GFP_ or empty vector. At approximately 24 h posttransfection, cells were infected with HCMV-TB40/E dark at an MOI of 1. At 48 hpi, TF_546_ was incubated with cells to label surface TFR. Cells were fixed 30 min following internalization of TF_546_. Internalized TF_546_ was detected via its own fluorescence (red). Endogenous EEA1 was indirectly visualized by labeling with anti-EEA1 rabbit antibody and secondary anti-rabbit Alexa Fluor 546 (green). TRE17_GFP_ was visualized via its own fluorescence (blue). Infection was confirmed by VAC morphology. A merged image with a higher-magnification inset of each channel is shown on the right of each panel. Download FIG S5, EPS file, 2.9 MB. Copyright © 2018 Zeltzer et al. 2018 Zeltzer et al. This content is distributed under the terms of the Creative Commons Attribution 4.0 International license.

**FIG 5:**
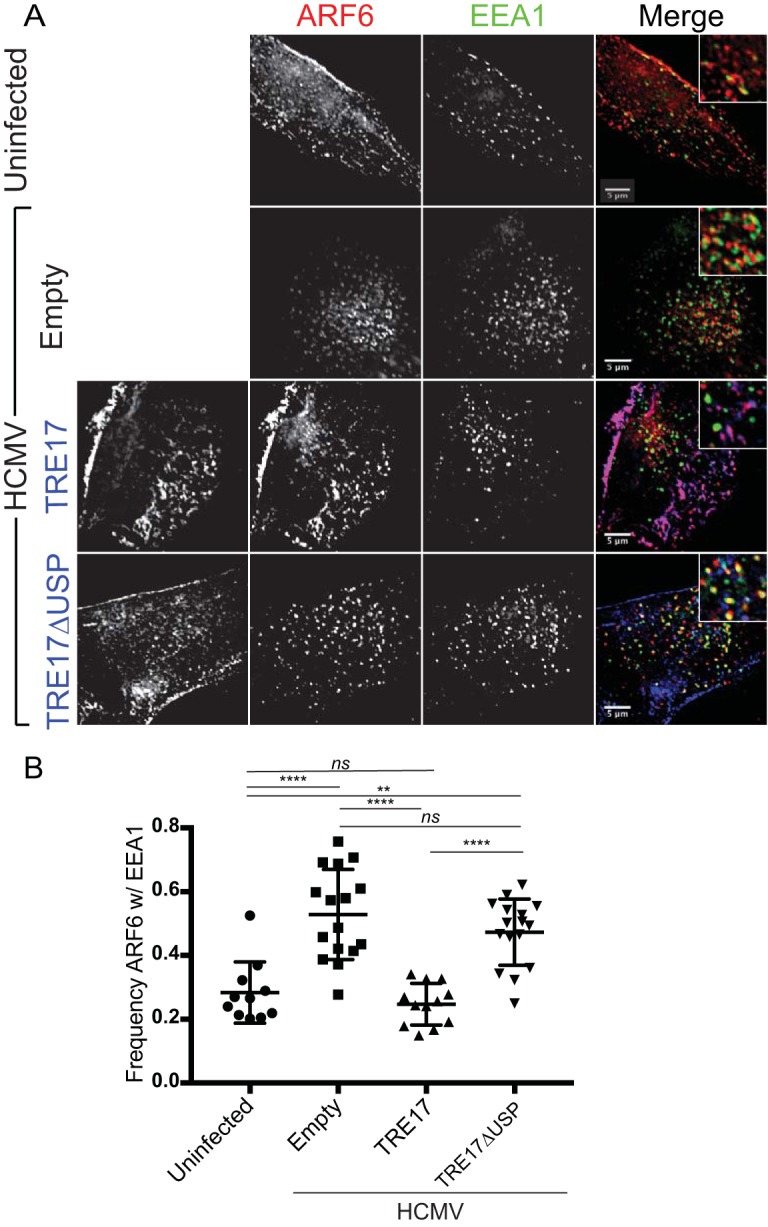
TRE17 induced ARF6-SE dissociation. Fibroblasts were transfected with plasmid expressing ARF6_mRuby_ and with TRE17_GFP_, TRE17ΔUSP_GFP_, or empty vector. At 24 h posttransfection, cells were mock infected or infected with HCMV-TB40/E dark at an MOI of 1. Cells were fixed at 48 hpi. (A) Endogenous EEA1 was indirectly visualized by labeling with anti-EEA1 rabbit antibody and secondary anti-rabbit Alexa Fluor 488 (green). ARF6_mRuby_ was visualized via its own fluorescence (red), and TRE17_GFP_ or TRE17ΔUSP_GFP_ was visualized via its own fluorescence (blue). Infection was confirmed by VAC morphology and IE2 staining (not shown). A merged image with a higher-magnification inset of each channel is shown on the right of each panel. Results of the experiments performed as described for panel A were imaged using a DeltaVision deconvolution microscope. Each image corresponds to the same representative focal plane. Bar, 5 µm. (B) Quantification of data from the experiments performed as described for panel A. Image quantification was performed by the use of the Squassh workflow method in the Mosaic suite of ImageJ and Fiji. The *y*-axis data represent frequencies of association of ARF6 with EEA1 and were calculated by hand-counting the number of plasma-membraned independent (vesicular) ARF6-positive structures separated by <1 px from EEA1-positive structures. Each value was divided by the total number of vesicular ARF6 to calculate the percentage of ARF6 associated with EEA1. Each dot represents an individual cell’s ARF6-EEA1 association frequency. The values in panel B represent means ± SD; an average of 14 cells were analyzed per condition. Statistical significance was determined by a one-way ANOVA with Tukey corrections. Asterisks (**, *P* = <0.01; ****, *P* = <0.0001) represent statistically significant differences.

Because the USP domain regulates the trafficking of CIE cargos such as MHC-I and CD98 to the PM (27, 30), we examined the effect of TRE17 overexpression on ARF6-EEA1 association in infected cells. Overexpression of TRE17_GFP_ decreased the association of ARF6_mRuby_ with EEA1 to a frequency similar to that observed for uninfected cells (Fig. 5). However, expression of TRE17ΔUSP had no effect on the ARF6-EEA1 association, suggesting that USP activity is required for TRE17-mediated dissociation of ARF6 membranes from SEs in infection. These results suggest that the USP activity of TRE17 is required for ARF6 dissociation from SEs.

### HCMV infection hijacks cargo sorting into EEA1 or ARF6 membranes in heterotypic SEs.

**FIG 6:**
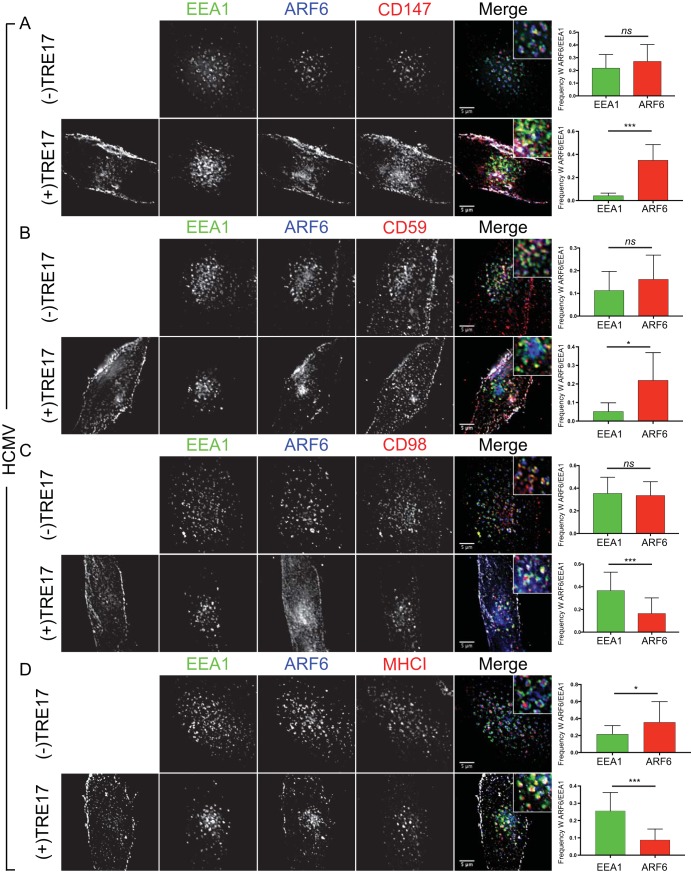
HCMV partitions CIE cargos into different membrane domains. Fibroblasts were transfected with plasmids expressing ARF6_mRuby_ and an empty vector [(−) TRE17 panels] or TRE17_GFP_ [(+)TRE17 panels]. At 24 h posttransfection, cells were infected with HCMV TB40/E dark at an MOI of 1. At 48 hpi, monoclonal mouse antibody against CD147 (A), CD59 (B), CD98 (C), or MHC-I (D) was incubated with cells to label surface proteins. Cells were fixed at 60 min following internalization of antibody/cargo. Internalized CIE cargo was detected with anti-mouse Alexa Fluor 647 (red). Endogenous EEA1 was indirectly visualized by labeling with anti-EEA1 rabbit antibody and secondary anti-rabbit Alexa Fluor 405 (green). TRE17_GFP_ was visualized via its own fluorescence (white). ARF6_mRuby_ was visualized via its own fluorescence (blue). Infection was confirmed by VAC morphology. A merged image with a higher-magnification inset of each channel is shown on the right of each panel. Results of the experiments represented in panels A to D were imaged using a DeltaVision deconvolution microscope. Each image corresponds to the same representative focal plane. Bar, 5 µm. Image quantification was performed by the use of the Squassh workflow method in the Mosaic suite of ImageJ and Fiji. The *y*-axis data represent the frequencies of coincidence of CIE cargo with EEA1 or ARF6 and are averages of results from three independent experiments. Quantifications of results from the experiments represented in panels A to D are shown to the right of the respective merged images. The values in panels A to D represent means ± SD; an average of 16 cells were analyzed per condition. Statistical significance was determined by a one-way ANOVA with Tukey corrections. Asterisks (*, *P* values = <0.05; ***, *P* values = <0.001) represent statistically significant differences.

Our finding that trafficking of the CIE mediator ARF6, but not of all CIE cargos, from EEA1 to the PM was restored by TRE17 was surprising. Therefore, we hypothesized that virus infection might differentially control CIE cargo sorting at the SE. If cargo such as MHC-I or CD98 was directed by infection into EEA1 membranes of a heterotypic SE, then it might be shielded or out of the reach of TRE17, which specifically associates with ARF6-GDP (27). To determine if CIE cargos are differentially sorted into different vesicular domains of the ARF6^+^/EEA1^−^ heterotypic SEs in HCMV infection, we exploited the ability of TRE17 to induce dissociation of ARF6 and EEA1 membranes into distinct populations. We transiently overexpressed TRE17_GFP_ or an empty vector with ARF6_mRuby_ in infected cells and analyzed the association of CIE cargos (CD147, CD59, CD98, and MHC-I) with either ARF6 or EEA1 following 60 min of antibody-induced uptake. In the absence of TRE17 overexpression in infected fibroblasts, all cargos were evenly distributed between EEA1 and ARF6, with the exception of MHC-I, which had a greater association with ARF6 than with EEA1 [Fig. 6, (−)TRE17 panels]. In infected cells overexpressing TRE17, both CD59 and CD147 were predominantly associated with EEA1^−^/ARF6^+^/TRE17^+^ membranes in HCMV infection [Fig. 6A and B, (+)TRE17 panels]. In contrast, MHC-I and CD98 were predominantly associated with EEA1^+^/ARF6^−^/TRE17^−^ vesicles [Fig. 6C and D, (+)TRE17 panels]. These results indicate that HCMV alters the sorting of CIE cargo into distinct membrane domains, which impacts the fate of these cargos—to recycle or be degraded.

## DISCUSSION

Endocytosis and vesicular trafficking are critical cellular processes that regulate cell surface composition, immune presentation, signaling, and the timely turnover of proteins to attenuate responses. Despite its pivotal role in regulating cell biology with implications for virus infection, very little is understood about how viruses modulate endocytosis beyond a means of virus entry into the host cell and maturation and egress of new progeny (34–36). In this study, we explored how HCMV regulates the immunologically relevant endocytic pathway, CIE. HCMV altered the endocytic itinerary of transport and immune proteins, such as CD147 and MHC-I, culminating in their long-term retention at SEs with the CDE cargo, TF. Further analysis determined that the major regulator of CIE, ARF6, is itself dysregulated by infection; ARF6 showed an increased association with SEs, resulting in enlarged ARF6-EEA1-positive structures. Therefore, we hypothesized that the retention of CIE cargo at SEs was due in large part to impaired ARF6-EEA1 membrane dissociation and recycling. We identified TRE17 as a host protein that, when overexpressed, restored ARF6/CIE trafficking from the SE. This work reveals a virus-induced block to trafficking from the SE where the virus controls decisions in endocytic sorting.

**FIG 7:**
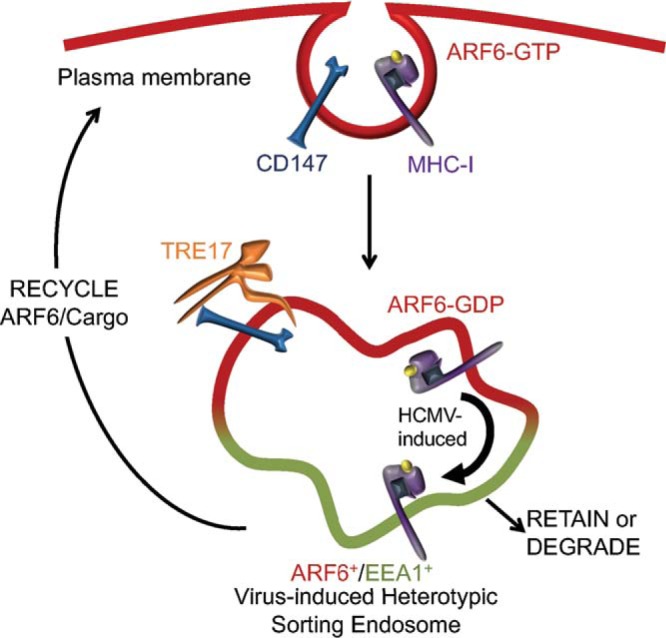
Model of CIE trafficking in the context of HCMV infection. CIE cargo was internalized through an AFR6-associated pathway and retained in EEA1^+^/AFR6^+^ sorting endosomes (the model presumes the occurrence of fusion). While these heterotypic endosomes are observed in uninfected cells, HCMV infection induces their accumulation, suggesting that they represent a normal but transient or infrequent part of cell biology. From our data, we propose that HCMV controls the sorting of cargo, particularly of MHC-I and CD98, between the ARF6 and EEA1 domains of the sorting endosomes. This has implications for receptor fate—to recycle, be retained, or degrade. Our work further demonstrates a role for the USP activity of TRE17 in allowing trafficking of ARF6 and its associated cargo, such as CD147 and CD59 from the sorting endosome.

Expression of TRE17, a ubiquitin-specific protease known for regulating both ARF6 and CIE cargo trafficking to the PM, rescued CD147 and, to a lesser extent, CD59 trafficking from SEs in the context of HCMV infection. Further, and in support of our model, overexpression of TRE17 in infection also decreased the ARF6-EEA1 association to the levels seen in uninfected cells and increased ARF6 concentration at the PM. The coincident staining of CD147 and CD59 with ARF6 at the PM suggests that their retention at the SE was indeed due in large part to impaired dissociation of ARF6/EEA1 membranes. Strikingly, the same was not true for MHC-I and CD98, which remained at EEA1^+^ SEs even in the presence of exogenous TRE17. Therefore, we propose a model whereby HCMV infection directs the sorting of cargo, such as MHC-I and CD98, from ARF6-positive domains into EEA1-positive domains in heterotypic SEs, which accumulate during infection (Fig. 7). Sorting cargo into EEA1-positive domains would presumably exclude cargo from the reach of TRE17 and thus prevent its trafficking with ARF6. In all, these observations are consistent with the notion that TRE17 USP activity is confined to conduits of the CIE pathway, namely, to proteins resident in ARF6-GDP membranes (27, 30). Taken together, these findings suggest that infection not only alters ARF6 dynamics and the associated cargos but also influences sorting events in a cargo-specific manner. While our studies did not seek to measure the implications of differential cargo sorting, we posit that rerouting MHC-I out of ARF6-positive membranes and into EEA1^+^ membranes may serve to encumber its recycling to the PM, thereby restricting antigen presentation. Indeed, it is possible that HCMV reprograms the fate of numerous cargos through migration into alternative endosomal microdomains, which may have profound effects on host signaling and response.

It is of interest to determine if HCMV infection alters TRE17 expression or activity. We detected an increase in TRE17 transcripts in infection (see Fig. S6 in the supplemental material), suggesting that there was no defect in expression of TRE17. However, due to the limitations of the tools available for TRE17, we were unable to determine if TRE17 protein levels or localizations were altered. TRE17 function is dependent on proper targeting, a process controlled by growth factor signaling and RAC1-mediated actin polymerization (37). Of note, RAC1 activation is regulated by ARF6 activation, a process we demonstrate to be dysregulated by infection (38). While HCMV infection may dysregulate TRE17, such that its overexpression restores ARF6 trafficking, it is equally possible that expression of TRE17 allows infected cells to overcome a defect in a highly related or interdependent component of the ARF6/CIE recycling system that has yet to be identified. We consider this scenario less likely since overexpression of related USPs does not affect CIE trafficking (30). Regardless, the effect of TRE17 on ARF6 trafficking in infection reveals a novel point of control in infection—the sorting of cargo between EEA1 and ARF6 domains.

10.1128/mBio.00683-18.6 FIG S6  Quantitative reverse transcription-PCR (qRT-PCR) analysis of TRE17. Fibroblasts were mock infected or infected with HCMV TB40/E at an MOI of 1. At the indicated time points (hours postinfection), RNA was isolated and cDNA synthesized. Following RT-qPCR, TRE17 transcripts were normalized to the housekeeping gene H6PD. Data represent means ± SD of technical triplicates from 2 representative experiments. Statistical significance was determined by a one-way ANOVA with Tukey corrections. Asterisks (*, *P* values = <0.05) represent statistically significant differences. Download FIG S6, EPS file, 1.8 MB. Copyright © 2018 Zeltzer et al. 2018 Zeltzer et al. This content is distributed under the terms of the Creative Commons Attribution 4.0 International license.

Previous studies in murine cytomegalovirus (MCMV) have reported the long-term retention of the CIE cargo MHC-I in endocytic pools (39). While those studies did not investigate any other CIE cargo or ARF6 trafficking, the results suggest that MCMV is dysregulating CIE in a manner similar to what we have described for HCMV. Further, Kaposi sarcoma-associated herpesvirus encodes two ubiquitin ligases (K3 and K5) that induce the rapid internalization of MHC-I; however, this form of CIE regulation appears to be MHC-I specific and linked to MHC-I degradation (40). Similarly, U21 of HHV-7 retains MHC-I at a juxtanuclear region and diverts MHC-I to the lysosome (41). However, we did not detect CIE cargo retention in the context of herpes simplex virus 1 (HSV-1) infection (Fig. S7), suggesting that this phenotype is shared across some but not all herpesviridae.

10.1128/mBio.00683-18.7 FIG S7  HSV-1 does not induce endosomal CD147 retention. Fibroblasts were mock infected or infected with HSV-1 at an MOI of 1. At 18 hpi, monoclonal antibody against CD147 was incubated with cells to label surface proteins. Cells were fixed 60 min following internalization of antibody/cargo. Internalized CD147 was detected with anti-mouse Alexa Fluor 647 (red). Endogenous EEA1 was indirectly visualized by labeling with anti-EEA1 rabbit antibody and secondary anti-rabbit Alexa Fluor 546 (green). HSV-1 infection was confirmed by detection of virally expressed GFP (not shown). A merged image with a higher-magnification inset of each channel is shown on the right of each panel. Results of experiments were imaged using a DeltaVision deconvolution microscope. Each image corresponds to the same representative focal plane. Bar, 5 µm. Download FIG S7, EPS file, 2.4 MB. Copyright © 2018 Zeltzer et al. 2018 Zeltzer et al. This content is distributed under the terms of the Creative Commons Attribution 4.0 International license.

Our analysis of ARF6 and EEA1 in both infected and uninfected cells revealed a common feature. ARF6 and EEA1 seldom showed any signs of integration; rather, the two proteins remained segregated but proximal. Membrane/protein partitioning of this sort has been described across the spectrum of endocytic trafficking and most likely confers specificity for distinct trafficking events (1, 42–47). In this scenario, the ARF6+ and EEA1+ domains would share a lumen (Fig. 2D, Fusion). It is also possible that ARF6 and EEA1, while proximal in infection, do not share a contiguous lumen (Fig. 2D, coupled); if such an event were the case, however, it would be difficult to imagine how a CIE cargo such as MHC-I ultimately partitions with EEA1. For this reason, we propose that the ARF6^+^/EEA1^+^ heterotypic vesicles represent a fusion where ARF6 and EEA1 are maintained in separate microdomains (Fig. 7).

The enlarged ARF6-EEA1-positive heterotypic endosomes generated by infection afforded us a unique opportunity to study trafficking and sorting events that are ordinarily transient and, therefore, difficult to monitor in uninfected cells. Perhaps the most fascinating observation afforded to us by this structure was a detailed analysis of TRE17’s effect on ARF6 and CIE cargo trafficking. Via its TRE-2/Bub2/CDC16 (TBC) domain, TRE17 preferentially interacts with ARF6-GDP at tubular endosomes and facilitates its recycling to the PM (27). Meanwhile, via a USP domain, TRE17 induces the trafficking of CIE cargo, such as MHC-I and CD98, to the cell surface (30). Our studies of TRE17 in infection married these distinct functions in two important ways. First, we demonstrated that TRE17 mediates the trafficking of CIE proteins, such as CD147, away from SEs as a consequence of either inducing ARF6 dissociation from SEs or preventing ARF6 association with SEs. Based on our observation of internalization of CD147 prior to TRE17-mediated trafficking (Fig. S3) and the preferential binding of TRE17 to ARF6-GDP (27), the former is more likely, as ARF6 undergoes GTP hydrolysis at SEs via interaction with the ACAP1/2 GAPs (26). Second, we showed that the ability of TRE17 to mediate ARF6 trafficking (and, therefore, CIE trafficking) is dependent on its USP activity, as expression of the TRE17 USP mutant in infection resulted in retention not only of ARF6 at the SE but also of its associated cargo. These observations marry the separate functions of the TBC and USP domains of TRE17. The TBC domain acts as an ARF6 anchor, priming TRE17 to traffic ARF6-GDP membranes back to the cell surface (27), while the USP domain acts as a switch, regulating the dissociation of ARF6 membranes from SEs. This dual-domain functionality operates similarly to an "AND" logic gate, ensuring that ARF6/cargo passage from the SE occurs only after two conditions have been realized: hydrolysis of ARF6-GTP to GDP (whereupon TRE17 can interact with ARF6) and protein deubiquitination. In line with this, the presence of a decreased concentration of ARF6-GTP in infected cells may lead to enhanced TRE17-mediated ARF6 activation. We posit that this ubiquitin cleavage step may serve as a sorting checkpoint to ensure that only deubiquitinated cargos return with ARF6 to the PM. To our knowledge, this represents the first time that vesicular dissociation has been shown to be regulated by deubiquitinase activity. This appears to be a novel mechanism for regulating both the timing and precision of endocytic sorting events and a method of control that may not be restricted exclusively to ARF6 trafficking or HCMV infection.

While numerous studies have highlighted altered endocytic organization following infection with HCMV, they mainly focused on the role that this reorganization plays in viral assembly and egress (2–4, 9, 48–50). While undoubtedly crucial, HCMV’s alteration of host trafficking results in an alternative series of events that appear to operate independently of vial assembly. Here we report that infection rewires the CIE pathway, culminating in the retention of numerous immune and transporter proteins in SEs within the viral assembly compartment. Previously, we found that phosphoactive EGFR is retained in Rab5- and Rab11a-positive endosomes in infection (8). Additional reports indicate a multitude of signaling and cargo proteins concentrated in a juxtanuclear region of infected cells (5, 7, 9, 10, 15). This pattern suggests one of two possibilities: (i) altered endocytosis is a consequence of viral assembly compartment formation or (ii) altered endocytic trafficking is a distinct event and, therefore, may confer an advantage for infection. We favor the latter argument for several reasons. Data from a time course of infection indicate that CIE retention begins no later than 24 h postinfection (Fig. S8A and B), well before the formation of the viral assembly compartment and after entry-associated endocytic events (2, 34). This is consistent with the observed localization of mTOR to a juxtanuclear region as early as 24 h following infection (51). These data, combined with our observation that particular cargos such as MHC-I appear to undergo infection-specific alternations in trafficking, suggest that the alteration to endocytic trafficking may serve a unique purpose for infected cells, altering antigen presentation as well as signaling. Cumulatively, the results of this study not only serve to describe a previously unappreciated means that HCMV employs to dysregulate host trafficking through perturbation of trafficking from the SE but also provide an in-depth molecular analysis of the CIE trafficking pathway and reveal novel functions for TRE17 as a dual regulator of the CIE/ARF6 sorting system in a ubiquitin protease-dependent manner.

10.1128/mBio.00683-18.8 FIG S8  Infection time course of CIE retention. (A) Fibroblasts were mock infected or infected with HCMV-TB40/E at an MOI of 1 for 60 min. Cells were washed of excess virus and returned to fresh media. At 11, 23, or 35 hpi, monoclonal mouse antibodies against CD98 were incubated with cells to label surface proteins. Cells were fixed at 60 min following internalization of antibody/cargo. Internalized CD98 was detected with anti-mouse Alexa Fluor 647 (red). Endogenous EEA1 was indirectly visualized by labeling with anti-EEA1 rabbit antibody and secondary anti-rabbit Alexa Fluor 546 (green). Infection was confirmed by detection of virally expressed GFP (not shown). A merged image with a higher-magnification inset of each channel with DAPI staining to indicate nuclei is shown on the right of each panel; hours postinfection are indicated to the left of corresponding panels. (B) Quantification of results from the experiment represented in panel A. Image quantification was performed by the use of the Squassh workflow method in the Mosaic suite of ImageJ and Fiji. The *y*-axis data represent the frequencies of coincidence of CD98 with EEA1. The values in panel B represent means ± SD; an average of 6 cells were analyzed per time point. Statistical significance was determined by a one-way ANOVA with Tukey corrections. Asterisks (**, *P* values = <0.01; ****, *P* values = <0.0001) indicate statistically significant differences. Download FIG S8, EPS file, 2.9 MB. Copyright © 2018 Zeltzer et al. 2018 Zeltzer et al. This content is distributed under the terms of the Creative Commons Attribution 4.0 International license.

## MATERIALS AND METHODS

### Cells and viruses.

Primary lung fibroblasts (MRC-5 and MRC9; ATCC), were maintained as previously described (52). TB40/E, a low-passage-number strain of human cytomegalovirus, was a gift from Christian Sinzger (53). TB40/E, a low-passage-number strain of human cytomegalovirus lacking GFP (TB40/E dark), was a gift from John Purdy. TB40/E expressing GFP as a marker for infection has been previously described (54). HSV-1 was a gift from Janko Nikolich-Zugich. All infections were conducted at a multiplicity of infection (MOI) of 1 in supplemented Dulbecco’s modified Eagle’s medium (DMEM) containing 10% fetal bovine serum (FBS) and viral inoculum. Mock-infected cells were provided supplemented DMEM containing 10% FBS only. Infection was monitored via GFP or, in the case of virus that did not express a fluorescent marker of infection, by IE2 expression or VAC morphology. Unless otherwise stated, all analysis was conducted on HCMV-infected cells at 48 h postinfection.

### Plasmids and transient transfections.

ARF6_mruby_ was created by digestion of ARF6 from ARF6_CFP addgene 11382 using Nhe1 and Age1, gel purification, and ligation upstream of mruby in pCIG. Rab11a GFP, Rab22a GFP, Rab35 GFP, ACAP1 Flag, Hook1 hemagglutinin (HA), TRE17 GFP, and TRE17C541S GFP were previously described (22, 26, 30, 33, 55). EHD3 GFP was a gift from Steve Caplan, University of Nebraska Medical Center. For transfection, actively dividing MRC5 or MRC9 cells were detached with trypsin, washed 3× in Dulbecco’s phosphate-buffered saline (PBS), and then suspended at 7.5 × 10^6^ cells per ml in Ingenio electroporation solution. One hundred microliters of cell suspension was mixed with 3 to 5 µg of DNA in 2-mm-path-length sterile cuvettes and electroporated using a Bio-Rad Gene Pulser Xcell system with a time constant at 130 V of 28 ms. Cells were then resuspended in supplemented media and plated for experiments. At approximately 12 to 16 h postelectroporation, cells were washed 3× in PBS and then mock infected or infected with HCMV at an MOI of 1.

### Antibody uptake assays and TF_546_ internalization assays.

Antibodies were resuspended in supplemented DMEM containing 10% FBS (see Table 1 for antibody concentrations). Coverslips containing cells were incubated with antibodies for 10 min at 37 C or 30 min at 37 C (MHC-I) unless otherwise specified. Coverslips were then washed three times in PBS and stripped of residual surface antibody by exposure to 0.5% acetic acid–5 mM NaCl at pH 3 for 30 s followed by three washes with PBS and returned to the media when appropriate. Following the desired internalization period, cells were washed three times in PBS and fixed for 20 min in 2% formaldehyde at room temperature. Postfixation, cells were washed three times in PBS and then permeabilized for 13 min in 0.1% Triton X-100–PBS. Slides were washed three times in PBS and blocked overnight in 0.5% bovine serum albumin (BSA)–5% normal goat serum–PBS. For TF_546_ endocytosis assays, mock-infected or HCMV-infected cells were provided TF_546_ at 100 µg/ml and allowed 10 min for internalization before being washed in PBS or (when coordinated with an uptake assay) an acid wash as described above. Unless otherwise stated, images were obtained using a DeltaVision RT inverted deconvolution microscope with a 60× objective. Representative single-plane images with 0.2-µm thickness were adjusted for brightness and contrast. SIM was conducted using a Zeiss Elyra S1 (SR-SIM) superresolution microscope and a Plan-Apochromat 63× objective. SIM images were rendered using ZEN imaging software.

**TABLE 1:** Antibodies and reagents used in this study^a^

Antigen	Antibody	Antibody type	Source	Concn	
				Immunoblotting	Immunofluorescence
ARF6	A305-238A	M	Bethyl	1:1,000^b^	
Alexa Fluor 546 TF	NA	NA	Thermo		100 µg/ml^f^
CD59	MEM-43/5	M	ABNova	ND	1:50^f^
CD98	MEM-108	M	BioLegend	ND	1:50^f^
CD147	HIM6	M	BioLegend	ND	1:50^f^
EEA1	C45B10	R	Cell Signaling	ND	1:100^c^
Flag epitope	M2	M	Sigma	ND	20 µg/ml^c^
HA epitope	6E2′	M	Cell Signaling	ND	1:1,600^c^
IE2	3H9	M	Gift^d^	ND	1:25^c^
MHC-I HLA B/C	HC-10	M	Gift^e^	1:50	ND
MHC-I HLA-A/B/C	W6/32	M	BioXCell	ND	1:50^f^
α-Tubulin	DM1A	M	Sigma	1:1,0000	

a NA, not applicable; ND, not done; R, rabbit; M, mouse.

b The dilution value represents dilution in Tris-buffered saline–5% milk supplemented with bovine serum albumin and Tween 20.

c The dilution value represents dilution in phosphate-buffered saline supplemented with bovine serum albumin and Tween 20.

d A generous gift from Tom Shenk, Princeton University.

e A generous gift from Lonnie Lybarger, University of Arizona.

f The dilution value represents dilution in complete medium.

### Image analysis.

Analysis of frequencies of coincidence was conducted using segmentation and quantification of the subcellular structures and the Squassh workflow method in the MosaicSuite for ImageJ and Fiji (56, 57). In brief, Z-stacks containing focal planes of intracellular signal in two channels were processed for analysis of the frequency of coincidence of one marker with another. ARF6-EEA1 coassociation analysis was conducted on segmented and background-subtracted images produced by the Squassh workflow. In brief, a representative focal plane was selected where counts were conducted. ARF6-EEA1 association was defined as any individual ARF6 region that was separated by <1 pixel (px) from an EEA1 region. These values were normalized to the total number of ARF6 regions counted per plane. ARF6 at the plasma membrane was excluded from this analysis. A total of 7 cells (on average) were counted for each of three biological replicates.

### Immunoblotting and ARF6 precipitation.

Immunoblotting was performed as previously described (58). Briefly, 25 to 50 µg of protein lysate was separated on 12% bis-Tris gels by electrophoresis and transferred to 0.45-µm-pore-size polyvinylidene difluoride membranes (Immobilon-FL; Millipore). Proteins were detected using epitope- or protein-specific antibodies and fluorescently conjugated secondary antibodies using an Odyssey infrared imaging system (Li-COR). All antibodies used are described in Table 1. ARF6 GTP pulldown experiments were performed using the ARF6 pulldown activation assay (Cytoskeleton, Inc.) and were conducted according to the instructions of the manufacturer (Cytoskeleton, Inc.). In brief, two 15-cm-diameter plates containing 6 × 10^6^ MRC5 cells (70% confluent) were incubated on ice, aspirated of media, and washed two times in ice-cold PBS. Next, ARF6 lysis buffer that included protease inhibitors was added to each plate before cells were scraped and collected. Samples were then centrifuged at 14,000 × *g* for 4 min at 4 C. The supernatant was then removed and snap-frozen in liquid nitrogen before storage at −80 C. Samples were then thawed and processed according to the manufacturer’s protocol. Whole-cell lysates were used to determine total ARF6 concentrations.

### Quantitative real-time PCR.

A total of 1 million primary lung fibroblast cells (either mock infected or infected with HCMV) were lysed in 400 µl of DNA/RNA lysis buffer (Zymo Research). RNA was isolated and cDNA was synthesized using a ZR Duet DNA/RNA Mini Prep kit according to the instructions of the manufacturer (Zymo Research). Primers for USP6/TRE17 (forward, CCTTCCAACCAGAGGGAGA; reverse, GCCTAAATGTAAGATGCCTCCA) were used to amplify USP6/TRE17 using Sybr green. TRE17 transcript levels were normalized to the cellular housekeeping gene, H6PD, which was amplified with the following primers: forward, GGACCATTACTTAGGCAAGCA; reverse, CACGGTCTCTTTCATGATGATCT.

Real-time PCR was carried out with a Roche LightCycler 480 system, and analysis was performed using Microsoft Excel.
